# Treatment of knee osteoarthritis with intra-articular injection of autologous adipose-derived mesenchymal progenitor cells: a prospective, randomized, double-blind, active-controlled, phase IIb clinical trial

**DOI:** 10.1186/s13287-019-1248-3

**Published:** 2019-05-21

**Authors:** Liangjing Lu, Chengxiang Dai, Zhongwen Zhang, Hui Du, Suke Li, Ping Ye, Qiong Fu, Li Zhang, Xiaojing Wu, Yuru Dong, Yang Song, Dongbao Zhao, Yafei Pang, Chunde Bao

**Affiliations:** 1 Department of Rheumatology, Ren Ji Hospital, School of Medicine, Shanghai Jiao Tong University, 145 Middle of Shandong Road, Huangpu District, Shanghai, 200001 People’s Republic of China; 2 Cellular Biomedicine Group, Shanghai, China; 3 Department of Orthopedics, The General Hospital of Chinese People’s Armed Police Forces, Beijing, China; 4 Department of MRI, The General Hospital of Chinese People’s Armed Police Forces, Beijing, China; 5 Department of Rheumatology, Changhai Hospital of Shanghai, Shanghai, China

**Keywords:** Intra-articular injection, Mesenchymal stem cells, Knee osteoarthritis, Magnetic resonance imaging, WOMAC

## Abstract

**Objective:**

Human adipose-derived mesenchymal progenitor cells (haMPCs) are stem cells with multiple differentiation potential and immunomodulatory function. Re-Join® comprises in vitro expanded haMPCs from adipose tissue of patients combined with cell suspension solution. This study was undertaken to evaluate the efficacy and safety of Re-Join® in patients with symptomatic knee osteoarthritis (OA).

**Methods:**

Patients with Kellgren–Lawrence grade 1–3 knee OA were recruited from two centers and randomized to receive intra-articular injection of Re-Join® or HA. Pain and function were assessed by using WOMAC score, VAS, and SF-36. Magnetic resonance imaging (MRI) analysis was performed to measure cartilage repair. Adverse events (AEs) were collected.

**Results:**

Fifty-three patients were randomized. Significant improvements in WOMAC, VAS, and SF-36 scores were observed in both groups at months 6 and 12 compared with baseline. Compared with the HA group, significantly more patients achieved 50% improvement of WOMAC and a trend of more patients achieved a 70% improvement rate in Re-Join® group after 12 months. Meanwhile, there was notably more increase in articular cartilage volume of both knees in the Re-Join® group than in the HA group after 12 months as measured by MRI. AEs were comparable between two groups. Most AEs were mild and moderate except one SAE of right knee joint infection in the HA group.

**Conclusions:**

Significant improvements in joint function, pain, quality of life, and cartilage regeneration were observed in Re-Join®-treated knee OA patients with good tolerance in a period of 12 months.

**Trial registration:**

ClinicalTrials.gov Identifier: NCT02162693. Registered 13 June 2014.

**Electronic supplementary material:**

The online version of this article (10.1186/s13287-019-1248-3) contains supplementary material, which is available to authorized users.

## Introduction

Around 9.6% of men and 18% of women aged over 60 years old have symptomatic osteoarthritis (OA) across the world [1]. The pathogenesis of OA is complex and not fully elucidated. Current treatments in early-stage OA include non-pharmacologic as well as pharmacologic therapy. Intra-articular injection of hyaluronic acid (HA) or platelet-rich plasma is also frequently used. However, disease-modifying therapies are still limited [2]. Disease progression to late-stage OA would eventually require joint replacement [3–5].

Mesenchymal progenitor cells (MPCs), or mesenchymal stem cells (MSCs), usually derived from umbilical cord blood, adipose tissue, or bone marrow, have been considered as potential therapeutic options for OA. By secreting a wide range of cytokines, MPCs have immunomodulatory functions that may skew the micro-environment of OA joints towards anti-inflammatory properties. Unlike direct cell engraftment and differentiation, MSCs could promote new cartilage-like cells in vitro [6], as well as boost repair and regeneration of cartilage and stimulate type II collagen production [7]. The efficacy of intra-articular injection of MSCs has been tested in several small randomized controlled studies [8], showing promising effects. Up to this point, more high-quality studies are needed to provide further evidence for autologous and allogeneic MPCs/MSCs in the treatment of OA.

Re-Join^®^ is a product composed of in vitro expanded autologous MPCs derived from adipose tissue of patients combined with cell suspension solution. Adipose tissue-derived MPCs were chosen because of easy and repeatable access to subcutaneous adipose tissue, simple isolation procedure, and high produce. Approximately 500-fold greater numbers of fresh MPCs can be derived from equivalent amounts of fat versus bone marrow [9, 10]. Our previous studies showed that Re-Join® was effective in animal models of OA in rabbit and sheep [11, 12]. Further dose-ranging phase I/IIa clinical trial suggested that Re-Join® was safe and effective in knee OA patients during 96 weeks of follow-up [13].

Here we conducted a randomized double-blind phase IIb clinical trial, evaluating clinical efficacy, cartilage imaging, and safety profile of intra-articular injection of Re-Join® with comparison of HA in patients with symptomatic knee OA.

## Methods

### Study design

The current study (registered at http://ClinicalTrials.gov with identifier: NCT02162693) was conducted between November 2013 and November 2016 at two clinical centers in the People’s Republic of China: Ren Ji Hospital, School of Medicine, Shanghai Jiao Tong University and The General Hospital of Chinese Armed Police. The study was conducted in accordance with the Good Clinical Practice (GCP) guidelines and the Declaration of Helsinki. The independent ethical committee at each center approved the protocol, and written informed consent form was obtained from all participants before screening.

### Inclusion/exclusion criteria

The study included patients who were between 18 and 70 years old, had a definite diagnosis of knee OA according to the American College of Rheumatology Clinical classification criteria for knee osteoarthritis and accompanied by pain in knee joint [14], and were below grade 4 by Kellgren–Lawrence criteria. Exclusion criteria included (1) history of allergy or allergic constitution; (2) concomitant severe infection, malignant tumor, coagulation disorder, or uncontrolled or unmanageable systemic diseases; (3) presence of other types of arthritis except OA; (4) intra-articular injection of HA or corticosteroid in the preceding 2 months; and (5) pregnant or breast-feeding women.

### Tissue and human adipose-derived mesenchymal progenitor cell (haMPC) processing procedure

We used the same standard operating process (SOP) as our phase I/IIa study and ISCT criteria for MSCs [13, 15]. Adipose tissue was obtained from abdominal subcutaneous by liposuction with local anesthetic. Isolation and culture of haMPCs were performed under Good Manufacturing Practice (GMP) conditions as previously described [13]. The haMPCs would not be released until passed all quality check including test for viability, population doublings, morphology, potency, identity, purity, and sterility.

### Randomization and intervention

All the patients enrolled were arranged to take liposuction, and autologous MPCs were prepared. Central randomization was performed by a biostatistician using PROC PLAN in SAS and executed in GMP workshop. Re-Join® or HA were shipped in a special vaccine box to research sites (temperature 4 to 8 °C) when patients needed therapy. Previous clinical trial results recommended 5 × 10^7^ haMPCs as the optimal administration dosage in the current study.

HA injections we used in the control arm were unified purchased and distributed. We chose ARTZ (ARTZ Dispo; 25 mg/2.5 mL; Seikagaku Corporation Japan) for the control arm. ARTZ is a 1% sodium hyaluronic acid (HA) that has been available on the Chinese market since 1997 and is widely used through intra-articular injection as an effective therapy for knee OA [16].

In order to maintain double blinding, the preparation for injection and the IA injection were performed in two different clean rooms by a trained experienced investigator, who was separate from the evaluator. A curtain was used to prevent patients from seeing the injection procedures. All study-related case report forms recorded only the randomization number.

In the HA group, intra-articular injection of HA was administrated once a week, four consecutive weeks (week 0, 1, 2, and 3). The haMPC group was injected with 5 × 10^7^ haMPCs (around 2.5 ml) at weeks 0 and 3. Sham injection was performed at weeks 1 and 2. Patients were advised to rest for 24 h following each injection.

### Assessments

Assessments were performed at screening, at baseline (prior to the first injection), 1 week after injection, and follow-up visits after 6 and 12 months.

The primary endpoint was the change of Western Ontario and McMaster Universities Osteoarthritis Index (WOMAC) score. The secondary endpoint included visual analogue scale (VAS), SF-36 questionnaire, magnetic resonance imaging (MRI) of knees, and safety profiles. Improvement rate was calculated for WOMAC, VAS, and SF-36, which was reported as the percentage of change of score in each time point of follow-up compared with baseline. Safety was assessed with adverse events (AEs) and serious adverse events (SAEs), electrocardiogram, vital signs, physical examination, and laboratory tests (including routine blood and urine tests, hepatic and renal functions tests, blood lipid and glucose tests, immunologic tests). Concomitant medications were recorded together with AEs and SAEs. All the detailed information for assessments was described in the previous study [13].

MRI evaluations were completed at screening and week 48. Knee cartilage volume (including the femur, tibia, and patella) was graded by two blinded, independent radiologists according to the methods described previously [17]. Details were described in Additional file 1.

### Statistics

Given the lack of safety and efficacy data of intra-articular haMPCs with other active comparator in patients with OA at the time of study design, the sample size was based on other MPC clinical trials for other indications.

The analyses presented were performed on the intent-to-treat (ITT) population. All data presented are on an ITT/last observation carried forward basis. A descriptive analysis, including anthropometric data, variables related to the medical history of patients, efficacy endpoints reported at baseline, and baseline laboratory parameters, was conducted. Number and percentages of patients who experienced AEs, SAEs, treatment-related AEs, and treatment-related SAEs were described by the treatment group. These values might be compared between the groups in the maintenance phase using a *χ*^2^ test or Fisher’s exact test. Other values were compared by Student’s *t* test or the Wilcoxon signed rank test according to the statistical distribution by normality test. Any change from baseline was presented as least squares mean estimates with 95% confidence intervals (CI); statistical significance was determined by a *P* value of &lt; 0.05. All statistical analysis was conducted with SAS software (V9.2, SAS Statistical Institute, Cart, NC, USA).

## Results

### Patient profiles

**Fig. 1:**
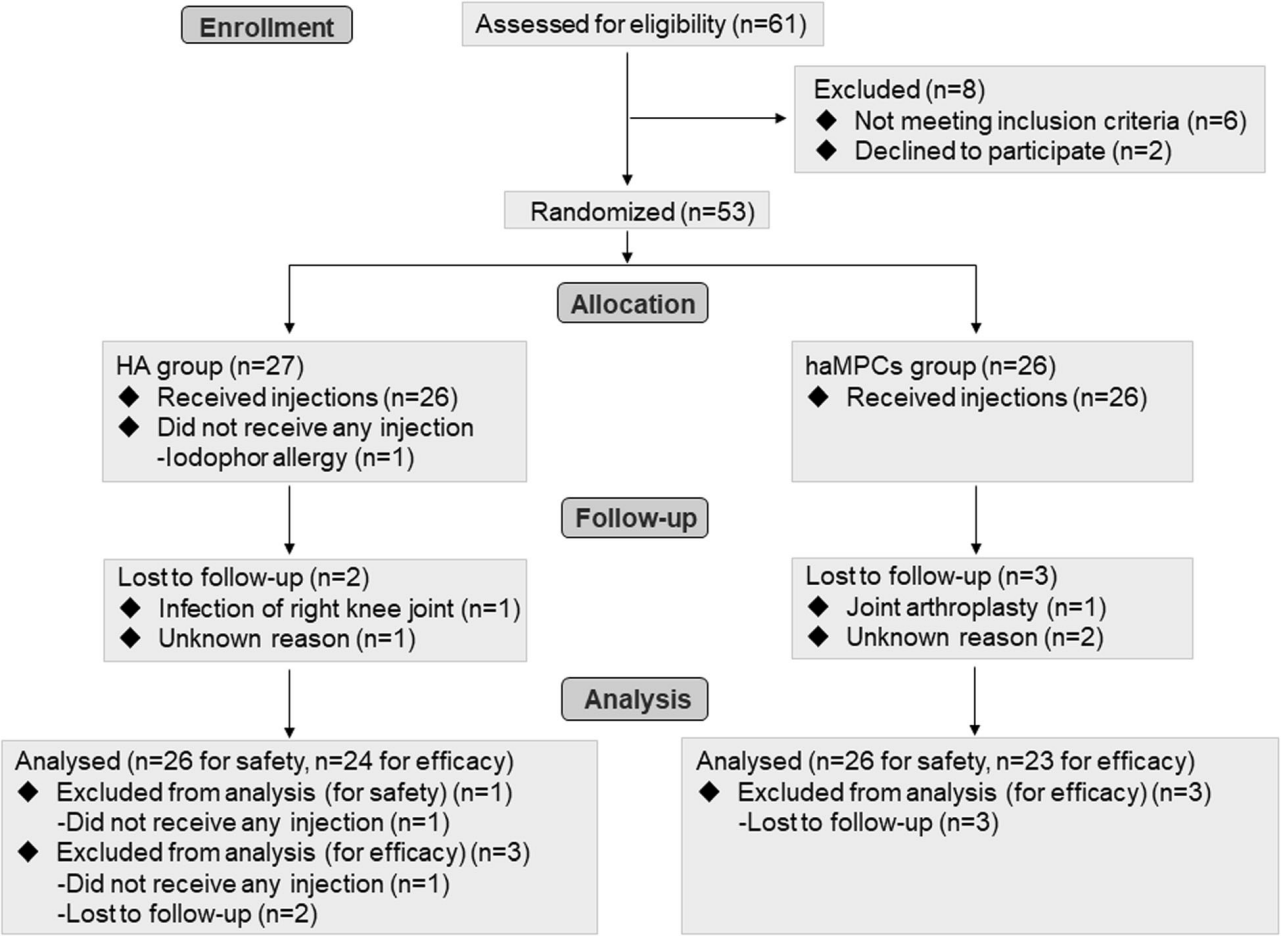
Flowchart of the clinical trial

The flowchart of the clinical trial is shown in Fig. 1. Among 61 patients screened, 53 patients were enrolled and randomized into two groups: 26 to the Re-Join® group and 27 to the HA group. One patient in the HA group did not receive treatment and withdrew from trial because of iodophor allergy during liposuction. Of the 52 participants, 47 (90.38%) completed the final study visit. Two patients in the HA group withdrew from the trial due to a case of injection associated right knee joint infection (described in safety profile) and an unknown reason, respectively. One patient in the Re-Join® group withdrew due to joint arthroplasty and two were lost to follow-up for unknown reasons.

Most patients enrolled were females aged about 55 years with an average body mass index (BMI) around 24 kg/m^2^. Patients in each group showed similar baseline characteristics in terms of height, weight, body mass index, radiographic grade of osteoarthritis, cartilage volume of both knees by MRI, previous treatment history, and concomitant disease, with a slightly younger age in the Re-Join® group than the HA group (Table 1).

**Table 1:** Clinical and demographic characteristics of the patients in the Re-Join® and HA groups (*n* = 52)

	Re-Join®		HA		*P* value	
No. of patients	26		26			
Age, mean (SD)	55.03 (9.19)		59.64 (5.97)		0.0375	
Sex, no. (%)						
Male	3 (11.54)		3 (11.54)		1.0000	
Female	23 (88.46)		23 (88.46)			
Height, mean (SD),cm	161.35 (6.43)		162.46 (5.66)		0.5609	
Weight, mean (SD), kg	63.46 (10.69)		62.81 (9.44)		0.8172	
Body mass index, mean (SD)	24.27 (3.04)		24.26 (2.59)		0.9883	
Symptom duration, mean (SD), month	53.62 (41.24)		63.81 (34.14)		0.2061	
Left knee VAS score, mean (SD)	5.27(2.27)		4.92(2.56)		0.6078	
Right knee VAS score, mean (SD)	5.50(2.48)		4.96(2.46)		0.4355	
Kellgren–Lawrence grade, No. (%)*	Left	Right	Left	Right	Left	Right
					0.825	0.825
Grade 0	0 (0.00)	0 (0.00)	0 (0.00)	0 (0.00)		
Grade 1	1 (3.85)	1 (3.85)	2 (7.69)	2 (7.69)		
Grade 2	9 (34.62)	9 (34.62)	8 (30.77)	8 (30.77)		
Grade 3	16 (61.54)	16 (61.54)	16(61.54)	16 (61.54)		
Grade 4	0 (0.00)	0 (0.00)	0 (0.00)	0 (0.00)		
Cartilage volume by MRI, log(mm^3^)	9.54 (0.19)	9.54 (0.18)	9.62 (0.19)	9.59 (0.19)	Left	Right
					0.124	0.347
Previous treatment, No. (%)^1^						
Yes	19 (73.08)		14 (53.85)		0.1539	
No	7 (26.92)		12 (46.15)			
Concomitant diseases, No. (%)^1^						
Yes	2 (7.69)		6 (23.08)		0.1279	
No	24 (92.31)		20 (76.92)			

*Statistics were calculated by ANOVA or chi-square test

### Clinical outcomes

**Fig. 2:**
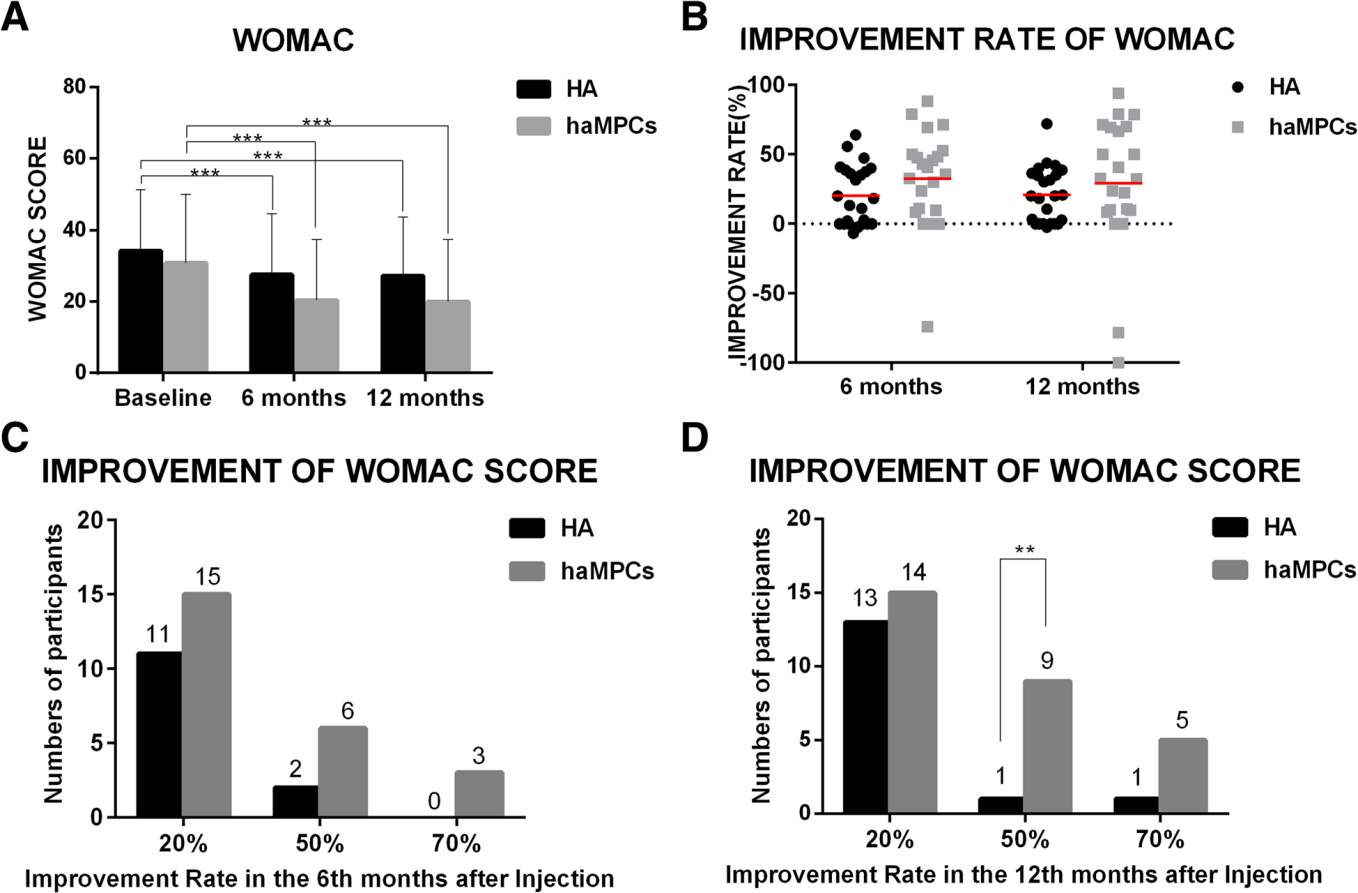
Changes of WOMAC score during 12 months after intra-articular injection of Re-Join® and HA. **a** Score and mean improvement rate of WOMAC, VAS, and SF-36 in the Re-Join® and HA groups at baseline and 6 months and 12 months after injection. **b** Mean improvement rate of WOMAC score compared with baseline in the Re-Join® and HA groups at baseline and 6 months and 12 months after injection. Statistics were determined by *t* test in **a** and **b**. **c**, **d** Number of patients who reached an improvement rate of 20%, 50%, and 70% according to the WOMAC score in 6 months (**c**) and 12 months (**d**) after injection compared with baseline. Score was shown in mean and standard deviation. Mean improvement rate was shown as the percentage of change of score in each time point of follow-up compared with baseline. Statistics were determined by *χ*^2^ test in **c** and **d**. *P &lt; 0.05, **P &lt; 0.01, ***P &lt; 0.001

A significant reduction of the WOMAC score was observed in both Re-Join® and HA groups in months 6 and 12 as compared to baselines, while the WOMAC change was similar between the two groups (*P* = 0.4753). Mean WOMAC score reduced from 30.83 ± 19.14 to 21.70 ± 17.87 (*P* = 0.0002) in the Re-Join® group and from 34.17 ± 17.16 to 27.58 ± 16.93 (*P* = 0.0001) in the HA group in month 6 after injection, showing an improvement rate of 31.65% and 20.23%, respectively. There was a trend of more reduction of WOMAC score in the Re-Join® group, but this difference did not reach statistical significance (*P* = 0.2197). In month 12, WOMAC score further reduced significantly to 21.35 ± 18.19 (28.52%, *P* = 0.0003) in the Re-Join® group and to 27.25 ± 16.33 (20.74%, *P* &lt; 0.0001) in the HA group. A slightly higher improvement rate was observed in the Re-Join® group compared to the HA group, but the difference was not statistically significant. (*P* = 0.2177) (Fig. 2a, b). When different subscales of WOMAC score were compared, we found that WOMAC pain, WOMAC stiffness, and WOMAC function scores reduced proportionally after treatment of Re-Join® and hyaluronic acid (Additional file 1: Table S1 and Figure S1).

When the participants of both groups were broken into subgroups according to the improvement rate of WOMAC score, more patients in the Re-Join® group reached 20%, 50%, and 70% of the improvement rate compared to those in the HA group 6 months after injection (15 vs. 11 in 20% subgroup, 6 vs. 2 in 50% subgroup, 3 vs. 0 in 70% subgroup), though the difference was not statistically significant. In month 12, similar numbers of patients could be seen in the 20% improvement rate subgroups for Re-Join® and HA (14 vs. 13, *P* = 0.6458). A significant larger number of participants could be seen in the 50% subgroup (9 vs. 1, *P* = 0.0038), but not in the 70% subgroup (5 vs. 1, *P* = 0.0742) in the Re-Join® group compared with the HA group (Fig. 2c, d). These data suggested that Re-Join® may have better long-term effects for OA patients.

**Fig. 3:**
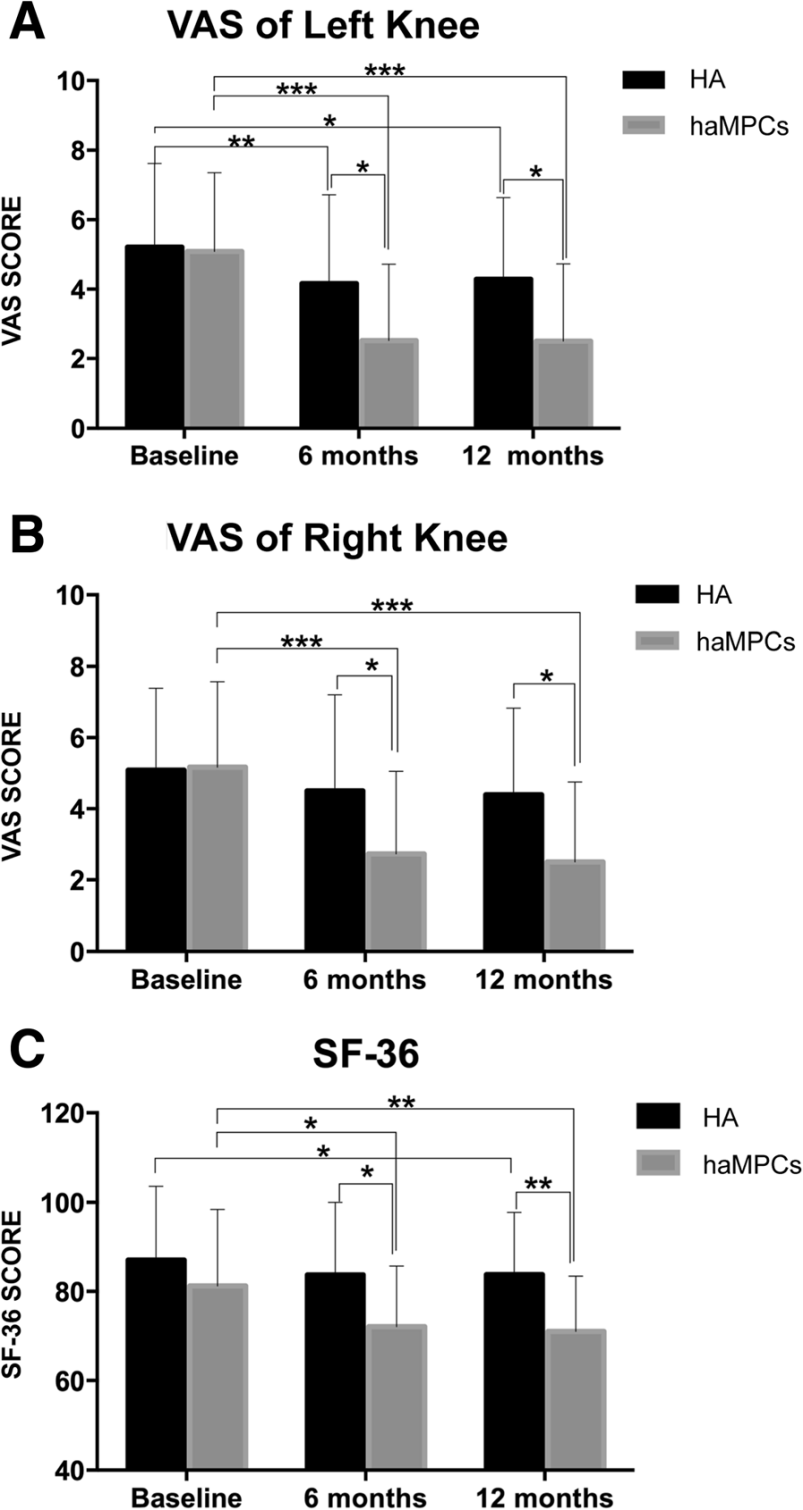
Changes of VAS and SF-36 scores during 12 months after intra-articular injection of Re-Join® and HA. **a** VAS score for the left knee in the Re-Join® and HA groups at baseline and 6 months and 12 months after injection. **b** VAS score for the right knee in the Re-Join® and HA groups at baseline and 6 months and 12 months after injection (**c**). SF-36 score in the Re-Join® and HA groups at baseline and 6 months and 12 months after injection. Data was shown in mean and standard deviation. Statistics were determined by *t* test. *P &lt; 0.05, **P &lt; 0.01, ***P &lt; 0.001

Both Re-Join® and HA were associated with reduction of VAS score during the follow-up. Significant reduction of VAS score could be observed in Re-Join® for both knees compared with HA in 6 months (2.85 ± 2.65 vs. 4.17 ± 2.55 with *P* = 0.0486 for the left knee and 3.00 ± 2.62 vs. 4.50 ± 2.71 with *P* = 0.0348 for right knee) and 12 months (2.83 ± 2.68 vs. 4.29 ± 2.35 with *P* = 0.0190 for the left knee, 2.78 ± 2.58 vs. 4.40 ± 2.43 with *P* = 0.0178 for right knee) (Fig. 3a, b).

For SF-36 score, a significant reduction could be observed in month 6 (from 81.35 ± 17.16 to 73.04 ± 14.16, *P* = 0.0113) and month 12 (from 81.35 ± 17.16 to 71.96 ± 12.79, *P* = 0.0031) in the Re-Join® group compared with the baseline. In the HA group, significance was observed in month 12 (from 87.04 ± 16.66 to 83.13 ± 15.59, *P* = 0.0481) but not in month 6 (from 87.04 ± 16.66 to 83.67 ± 16.46, *P* = 0.0874). When comparing the Re-Join® group with the HA group, significant reduction could be seen in the Re-Join® group both at month 6 (73.04 ± 14.16 vs. 83.67 ± 16.46, *P* = 0.0161) and month 12 (71.96 ± 12.79 vs. 83.13 ± 15.59, *P* = 0.0097) (Fig. 3c). These results showed that Re-Join® could effectively improve quality of life for OA patients.

### Radiological outcomes

**Fig. 4:**
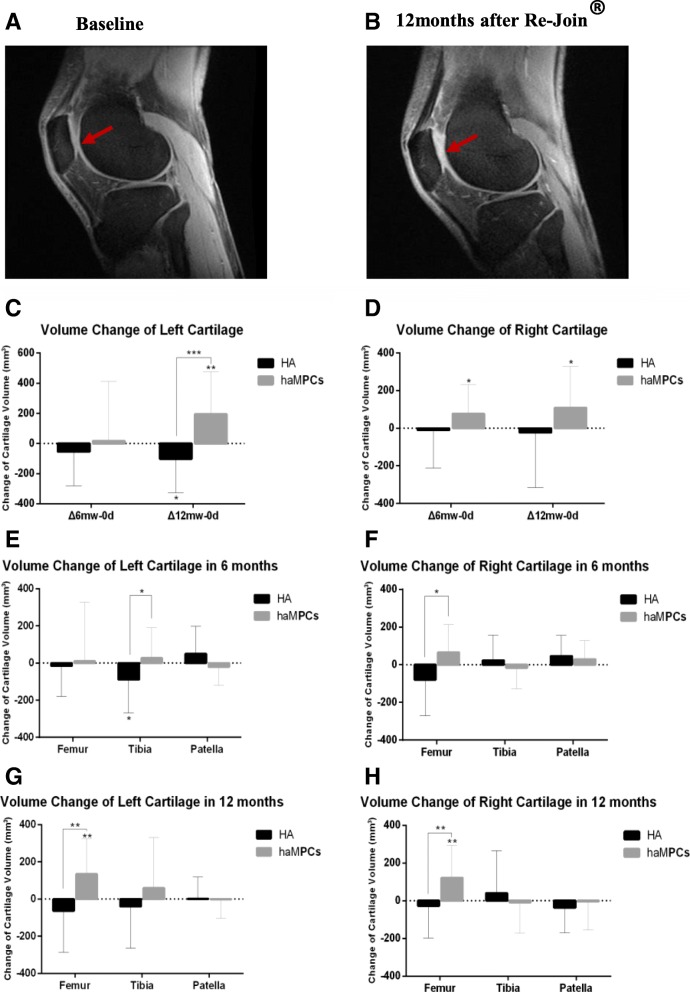
Changes of articular cartilage volume by MRI during 12 months after intra-articular injection of Re-Join® and HA. **a**, **b** Representative MRI images of the knee joint before and after treatment with Re-Join®. Sagittal views at the height of the patella-femoral condyles before and after 12 months of treatment. The arrows indicate the zones in which treatment generated a mild change in terms of cartilage thickness. **c** Changes of left knee cartilage volume by MRI in the Re-Join^@^ and HA groups at baseline and 6 months and 12 months after injection. **d** Change of right knee cartilage volume by MRI in the Re-Join^@^ and HA groups at baseline and 6 months and 12 months after injection. **e** Changes of left knee cartilage volume by MRI of different anatomy location in 6 months after injection. **f** Changes of right knee cartilage volume by MRI of different anatomy location in 6 months after injection. **g** Changes of left knee cartilage volume by MRI of different anatomy location in 12 months after injection. **h** Changes of right knee cartilage volume by MRI of different anatomy location in 12 months after injection. Data was shown in mean and standard deviation. Statistics were determined by Wilcoxon signed rank test. Baseline was shown as 0d, changes of knee cartilage volume were shown as Δ6mw-0d and Δ12m-0d. *P &lt; 0.05, **P &lt; 0.01, ***P &lt; 0.001

An increase in articular cartilage volume of both knees could be observed after Re-Join® therapy by MRI. Representative MRI images are shown in Fig. 4a and b. In month 6 after injection, the total volume of articular cartilage increased by 17.25 ± 394.23 mm^3^ (*P* = 0.8431) compared with the baseline for the left knee and 77.81 ± 155.37 mm^3^ (*P* = 0.0327) for the right knee. In month 12, a significant increase was found for the left knee [193.36 ± 282.80 mm^3^ (*P* = 0.0042)] and for the right knee [108.70 ± 220.13 mm^3^ (*P* = 0.0307)]. For the HA group, no significant increase but a decrease tendency was observed in the volume of cartilage during a 12-month follow-up, with a change of cartilage volume by − 54.00 ± 227.21 mm^3^ (*P* = 0.2666) for the left knee and − 10.15 ± 201.59 mm^3^ (*P* = 0.8115) for the right knee in month 6, and by − 101.88 ± 224.30 mm^3^ (*P* = 0.0362) for the left knee and − 23.47 ± 291.37 mm^3^ (*P* = 0.6967) for the right knee in month 12 (Fig. 4c, d).

To investigate the impact of Re-Join® on different anatomical locus of knee cartilage, the volume was measured and calculated separately by femur, tibia, and patella (Fig. 4e–h). Similar with the total cartilage, HA was not associated with significant increased cartilage volume of femur, tibia, and patella. Overall, a tendency of decrease was observed for femur and tibia, and a significant decrease was found in month 6 for the left tibia (change volume of − 88.95 ± 179.13 mm^3^, *P* = 0.0263). Compared with HA, injection of Re-Join® was associated with a significant increase in femur cartilage both left (− 63.50 ± 222.71 mm^3^ vs. 134.63 ± 189.16 mm^3^, *P* = 0.0086) and right (− 26.71 ± 170.69 mm^3^ vs. 121.36 ± 172.25 mm^3^, *P* = 0.0038) at month 12.

### Safety

All the 52 patients completed follow-up for safety assessment. No death occurred and no significant change was found from the results of electrocardiogram, vital signs, physical examination, and laboratory tests during the 12 months of follow-up. Adverse events occurred in a similar proportion between the two groups with 53.85% in the HA group and 73.07% in the Re-Join® group (*P* = 0.1144). The most common adverse events were transient pain and swelling of injection-site joint, all of which were mild to moderate and were spontaneously relieved within 7 days without special treatment. One SAE (1.92%) occurred in the HA group and the patient endured infection of right knee joint after 2 months of first injection and the patient withdrew from the study. This SAE was relieved after articular cavity flushing operation. No SAE occurred in the Re-Join® group during 12 months of follow-up (Table 2).

**Table 2:** Adverse and severe adverse events of OA patients after Re-Join® and HA treatment (*n* = 52)

Group	Number of patients	Number of injections	AEs, *n*	SAEs, *n*	Frequency of AE (%)	Frequency of SAEs (%)	*P* value
HA	26	104	14	1	53.85	3.846	0.1144
Re-Join^@^	26	103	19	0	73.07	0	
Total	52	207	33	1	63.46	1.92	

*AE* adverse event, *SAE* serious adverse events

## Discussion

HaMPCs were first discovered and identified in the early 2000s and have been shown to possess self-renewal capacity and multilineage differentiation potential [11, 18, 19].

HaMPCs have several advantages including easier and faster expansion in culture, more passage cells which still retain stem cell phenotypes and pluripotency, less susceptibility to age, and less morbidity of patients [9, 20, 21]. Despite all those advantages of haMPCs, data are limited regarding the effects of direct injection of haMPCs into the knees of OA patients [22–25]. Our phase I/IIa clinical trial conducted has demonstrated the safety and optimal dosage of haMPCs for intra-articular injection in OA patients [13]. However, no clinical trials with the control group have been performed, nor comparison of therapeutic effects between haMPCs and other reported effective medicine by intra-articular injection.

Injection of HA was reported as a safe and well-tolerated treatment for osteoarthritis of the knee and other joint diseases, with a low incidence of side effects [14, 26, 27]. It is also reported that HA may restore the damaged HA layer on the articular cartilage surface and bring about an alleviation of the arthritic condition and an arrest of the progress of the disease [27]. HA was reported to be effective in reducing inflammation and protecting articular cartilage and be beneficial in patients with knee OA; therefore, it is widely used in knee OA treatment [14, 28, 29]. Hence, haMPCs were compared with HA in this phase IIb trial.

Re-Join® was superior to HA in terms of pain relief and improvement of quality of life as was shown by VAS and SF-36 in the “Results” section. Both the Re-Join® and HA groups showed a significant reduction of WOMAC after months 6 and 12 from baseline. Injection of Re-Join® showed a trend of a higher improvement rate compared with HA although the difference was not statistically significant. The main reason was probably because of a small sample size and heterogeneity of response. Two patients in the Re-Join® group did not respond to the injection and the WOMAC score gradually increased during the follow-up, causing a significant drop-off in the improvement rate as shown in Fig. 1b. Removal of the two patients leads to a statistically significant increase both in month 6 (*P* = 0.0238) and month 12 (*P* = 0.0233) (data not shown). Further researches were needed to explore the reason for the heterogeneity of patients’ response.

It was interesting to find intra-articular injections of Re-Join® had an effect on the increase of cartilage volume, with a prominent increase 12 months after injection. This effect is mainly shown on femur. While in the HA group, a decrease of cartilage could be observed in the total volume, femur, tibia, and patella, much like the natural course of progression in OA. The effect of cartilage repair by Re-Join® was long-acting, which was consistent with its regeneration potential and might provide promising therapeutic intervention and cartilage repair for OA patients.

The Re-Join® group had comparable AEs and treatment-related AEs (TEAEs) with the HA group. Incidence of AEs in HA and Re-Join® was consistent with similar knee HA trials [14, 30] and haMPC trial for OA [22]. One SAE, an intra-articular infection in the HA group, was considered unrelated to treatment because the infection occurred 2 months later after injection, although this SAE resulted in withdrawal of the patient.

There are some limitations of the study. First, it was a study with a relatively small sample size. Second, patients enrolled in this study were all below grade 4 by Kellgren–Lawrence grade. Whether Re-Join® could be effective in patients with more severe OA was not known and needs further studies. Third, while regeneration of articular cartilage was clearly identified with MRI, the duration of therapeutic effect of Re-Join® is still unknown. In the circumstance that OA is a chronic and progressive disease and the effect of HA is relatively short to medium term [31, 32], this is a matter of concern. We will continue following up these patients and data with a longer period might provide more evidence.

## Conclusion

In summary, Re-Join® could improve function, pain of knee, quality of life, and cartilage regeneration in this randomized double-blind controlled study. These results, together with our previous preclinical OA animal model study and phase I/IIa clinical trial, support promising therapeutic potential of intra-articular injection of adipose tissue-derived MPCs in the treatment of knee OA. More studies with a larger sample size and with heterogeneous MPCs are warranted to further evaluate the efficacy and safety profile of Re-Join® in OA patients.

## Additional file

Additional file 1: Supplemental MRI method and WOMAC subscales score. (DOCX 103 kb)

## Abbreviations

AEs: Adverse events
BMI: Body mass index
CI: Confidence intervals
GCP: Good Clinical Practice
GMP: Good Manufacturing Practice
HA: Hyaluronic acid
haMPCs: Human adipose-derived mesenchymal progenitor cells
ITT: Intent to treat
MPCs: Mesenchymal progenitor cells
MRI: Magnetic resonance imaging
MSCs: Mesenchymal stem cells
OA: Osteoarthritis
SAEs: Serious adverse events
SOP: Standard operating process
VAS: Visual analogue scale
WOMAC: Western Ontario and McMaster Universities Osteoarthritis Index
