# Supplementary material for: Treatment of knee osteoarthritis with intra-articular injection of autologous adipose-derived mesenchymal progenitor cells: a prospective, randomized, double-blind, active-controlled, phase IIb clinical trial
Source: Stem Cell Res Ther. 2019 May 21;10:143. doi: 10.1186/s13287-019-1248-3 (PMC6528322; doi:10.1186/s13287-019-1248-3)
Supplement: Supplementary file 1 — Supplemental MRI method and WOMAC subscales score. (DOCX 103 kb) [file 13287_2019_1248_MOESM1_ESM.docx]

Additional file 1

1. Supplementary methods:

Knee MRIs were obtained with a 16-channel knee coil on a 3.0T MRI machine using a standard diagnostic protocol (resolution1.2 mm, RadCore/BioTelemetry). Cartilage volume measurements were made using a 3D SPGR with fat suppression sequence, oblique sagittal orientation, 110 slices, repetition time 13.2ms, echo time 4.5ms, flip angle 15°, and 0.56x0.46x0.60 mm^3^ voxel resolution. MRI evaluations were completed at screening and week 48. All imaging was standardized with study-specific imaging acquisition guidelines.

Knee cartilage volume (including the femur, tibia and patella) was graded by two blinded, independent radiologists according to the methods of Bae et al, a semi-automated segmentation method using a graph-cuts algorithm^[1]^. The first step included the manual placement of initial seeds to segment and indicate cartilage vs. non-cartilage. Then, an automated computational segmentation of the cartilage occurred using a graphs cut-algorithm^[2]^. When the segmentation was not satisfactory, the radiologist repeated these steps to accurately quantify the cartilage volume as a whole and into three distinct compartments: femur, tibia and patella.

In addition, the two experienced radiologist performed semiquantitative scoring of the fat-suppressed sequences using a modified Whole Organ Magnetic Resonance Imaging Score (WORMS)^[3]^.

1. WOMAC pain, stiffness and function scores at baseline and after treatments.

Table S1.
WOMAC subscales score at baseline as well as 6 and 12 months afterwards.

| WOMAC | Time | Re-Join^®^ | HA | P vale |
| --- | --- | --- | --- | --- |
| Pain | Baseline | 7.69(4.08) | 7.23(3.68) | 0.6701 |
|  | 6 months | 5.08(3.10) | 5.88(3.57) | 0.3948 |
|  | 12 months | 4.75(3.44) | 5.92(3.38) | 0.1774 |
|  | Δ, 6 months | -2.62(2.21) ** | -1.48(1.53) ** | 0.0278 |
|  | Δ, 12 months | -2.63(2.36) ** | -1.44(1.85) ** | 0.0323 |
| Stiffness | Baseline | 2.42(1.94) | 2.58(1.79) | 0.7346 |
|  | 6 months | 1.73(1.71) | 2.08(1.80) | 0.4772 |
|  | 12 months | 1.63(1.64) | 2.16(1.84) | 0.3058 |
|  | Δ, 6 months | -0.69(1.49)* | -0.52(1.26) | 0.5091 |
|  | Δ, 12 months | -0.67(1.61)* | -0.44(1.26) | 0.3587 |
| Function | Baseline | 23.65(14.60) | 22.35(13.29) | 0.7369 |
|  | 6 months | 17.00(13.40) | 18.52(12.85) | 0.6171 |
|  | 12 months | 15.67(13.38) | 18.20(12.23) | 0.3265 |
|  | Δ, 6 months | -6.65(7.11)** | -4.32(7.24)** | 0.2538 |
|  | Δ, 12 months | -7.04(8.06)** | -4.64(6.41)** | 0.2072 |
| Overall | Baseline | 33.77(19.99) | 32.15(18.07) | 0.9343 |
|  | 6 months | 23.81(17.82) | 26.48(17.47) | 0.5913 |
|  | 12 months | 22.04(18.12) | 26.28(16.71) | 0.2417 |
|  | Δ, 6 months | -9.96(9.97)** | -6.32(7.96)** | 0.1480 |
|  | Δ, 12 months | -10.33(11.18)** | -6.52(7.25)** | 0.1189 |

The values of each one of the three WOMAC subscales as well as the overall WOMAC score at baseline and 6 and 12 months afterwards are presented. Data are the mean (SD) of each group. Function means physical function. Δ represents the mean change from baseline (at 6 months and 12 months) in the OA patients for clinical outcome parameters. * p<0.05, ** p<0.01 with respect to the baseline value of the same group.


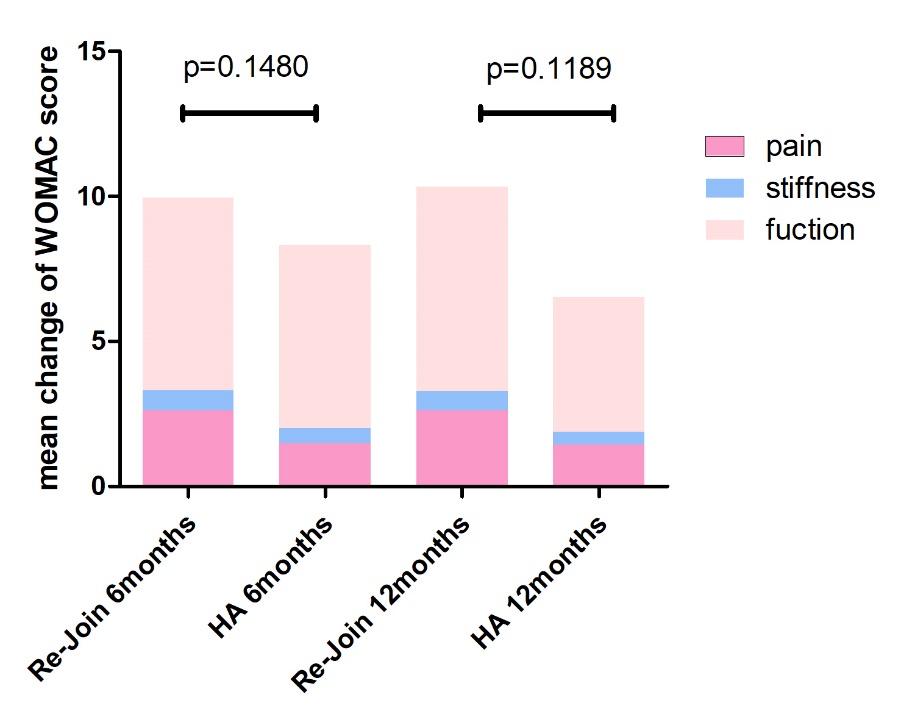


1. Figure S1. Contribution of WOMAC subscales change in the change of total WOMAC score 6 and 12 months after treatment of Re-Join® or Hyaluronic acid.

reference

1. Bae KT, Shim H, Tao C, et al. Intra- and inter-observer reproducibility of volume measurement of knee cartilage segmented from the OAI MR image set using a novel semi-automated segmentation method. Osteoarthritis Cartilage 2009;17:1589-97.
2. Boykov Y, Funka-Lea G. Graph cuts and efficient ND image segmentation. International journal of computer vision 2006;70:109-31.
3. Peterfy C, Guermazi A, Zaim S, et al. Whole-organ magnetic resonance imaging score (WORMS) of the knee in osteoarthritis. Osteoarthritis and Cartilage 2004;12:177-90.
